# Nonsurgical Endodontic Management of an Odontogenic Cutaneous Sinus Tract in a Child: A Case Report

**DOI:** 10.3390/children13070882

**Published:** 2026-06-30

**Authors:** Ralitsa Bogovska-Gigova, Maria Kirilova

**Affiliations:** Department of Pediatric Dentistry, Faculty of Dental Medicine, Medical University Sofia, 1431 Sofia, Bulgaria; m.kirilova@fdm.mu-sofia.bg

**Keywords:** odontogenic cutaneous sinus tract, chronic apical periodontitis, nonsurgical endodontic treatment, pediatric endodontics

## Abstract

**Highlights:**

**What are the main findings?**

- Nonsurgical root canal treatment alone led to complete resolution of an odontogenic cutaneous sinus tract in a pediatric patient without surgical intervention.
- Elimination of the intracanal infection resulted in progressive periapical healing and spontaneous closure of the extraoral lesion within 6 months.

**What are the implications of the main findings?**

- Accurate diagnosis of cutaneous facial lesions of dental origin is critical to avoid mismanagement and unnecessary surgical or dermatologic treatments.
- Conservative endodontic therapy should be considered the first-line approach for managing odontogenic sinus tracts in children to preserve tooth structure and prevent scarring.

**Abstract:**

Odontogenic cutaneous sinus tracts represent an uncommon clinical manifestation of chronic dental infection and are frequently misdiagnosed due to the absence of dental symptoms and their resemblance to dermatologic lesions. This case report describes the nonsurgical endodontic management of a cutaneous sinus tract of dental origin in a 13-year-old patient. The patient presented with a persistent extraoral lesion in the mandibular region, initially evaluated by non-dental specialists. Clinical and radiographic examination revealed a necrotic mandibular first molar associated with a periapical radiolucency and intraoral sinus tract. Nonsurgical root canal treatment was performed using chemomechanical debridement with sodium hypochlorite irrigation and calcium hydroxide as an intracanal medicament. Complete obturation was achieved following resolution of intracanal exudation. No surgical intervention of the cutaneous lesion was undertaken. Progressive healing of the periapical lesion and spontaneous resolution of the extraoral sinus tract were observed over a 6-month follow-up period. This case is noteworthy because it combines a prolonged diagnostic delay, an atypical extraoral manifestation in a child, and successful resolution by nonsurgical endodontic therapy alone without surgical excision of the cutaneous lesion. The 6-month follow-up confirms sustained clinical and radiographic healing, underscoring the importance of early recognition and conservative management in pediatric patients. Early identification and elimination of the dental source can prevent unnecessary surgical procedures and minimize the risk of permanent scarring.

## 1. Introduction

Caries lesions are areas of localized destruction of dental hard tissues caused by acidic by-products from bacterial fermentation of dietary sugars within the dental plaque biofilm [1]. The process is dynamic, involving alternating periods of demineralization and remineralization, with net mineral loss resulting in lesion formation [2]. The development of dental caries is multifactorial. Acidogenic and aciduric bacteria metabolize fermentable carbohydrates, producing acids that lower the pH at the tooth surface [3]. When the pH drops below a critical threshold, demineralization occurs. Saliva acts as a buffer and provides calcium and phosphate for remineralization, while fluoride catalyzes remineralization and increases resistance to acid attack [4]. The disease progresses from subclinical molecular changes to visible cavitation, and can affect both coronal and root surfaces [5].

Complications of untreated dental caries include pain, pulpitis, pulpal necrosis, periapical infection, and potential systemic spread of oral bacteria, leading to sepsis or involvement of distant organs [1]. Advanced caries can result in tooth loss, abscess formation, and, in rare cases, life-threatening infections such as facial cellulitis or systemic sepsis [6,7].

In immature teeth in children, dental caries develop rapidly due to several unique anatomical and biological factors. Immature roots are characterized by thin dentin walls, wide open apices, and incomplete root formation, making them more susceptible to rapid carious progression and pulpal involvement [8]. Primary and newly erupted permanent teeth with less mineralized enamel and dentin are more vulnerable to acid dissolution and rapid caries extension toward the pulp [8]. This increased susceptibility is due to the lower mineral content and higher porosity of immature enamel and dentin, which facilitates faster acid penetration and demineralization [9]. Developmental enamel defects, such as hypoplasia, further increase caries risk by creating thinner, more plaque-retentive, and acid-sensitive surfaces [10].

Teeth with immature roots in children experience a higher frequency and more rapid progression of complications such as pulpitis, pulpal necrosis, periapical periodontitis, and dental abscesses. Histopathologic studies demonstrate that deep caries in immature teeth rapidly lead to severe pulp inflammation, necrosis, and significant alterations in apical tissues, including the apical papilla and Hertwig’s epithelial root sheath [11]. Pulpal involvement in immature permanent teeth poses unique clinical challenges owing to their distinctive anatomical and developmental characteristics [12]. These teeth typically present with open apices, thin dentinal walls, and incomplete root formation, rendering them particularly vulnerable to rapid progression of pulpal pathology and limiting the feasibility of conventional endodontic techniques [13]. In this context, preservation of pulp vitality is paramount, as it enables continued root maturation and apical closure—critical determinants of long-term tooth survival and functional integrity [12,13,14].

When the pulp remains vital or exhibits only reversible pulpitis, vital pulp therapy represents the preferred initial strategy. Options include indirect pulp treatment through selective caries removal (strongly recommended for deep lesions with a normal or reversibly inflamed pulp), direct pulp capping for small, controlled exposures, partial pulpotomy (removal of a limited amount of inflamed tissue), and full pulpotomy for more extensive involvement [15,16,17,18,19]. Clinical decision-making should therefore be guided by accurate assessment of pulp status, root maturity, and individual patient factors.

A facial sinus tract in children is an abnormal channel that forms between a site of chronic dental infection (a periapical or periodontal abscess) and the skin surface of the face. This tract allows purulent material to drain externally, often presenting as a persistent or intermittently draining nodule or ulcer on the facial skin, most commonly in the mandibular or submandibular region in children [20,21,22].

A facial sinus tract occurs when chronic infection from a necrotic pulp or advanced periodontitis leads to suppurative inflammation and bone destruction. The infection follows the path of least resistance, perforating the cortical bone and soft tissues, eventually reaching the skin surface. This process is more likely when intraoral drainage is impeded or when the infection is long-standing and inadequately treated. In children, delayed diagnosis is common due to the absence of dental symptoms, and the lesion may be misdiagnosed as a dermatologic condition [23,24]. The development of a facial sinus tract is a sign of chronicity and indicates that the underlying dental infection has breached the alveolar bone and soft tissue barriers. Prompt recognition and elimination of the dental source—typically by root canal therapy or extraction—results in resolution of the tract. Failure to address the dental etiology can lead to persistent infection, scarring, or more severe complications such as cellulitis or osteomyelitis [20,21,25].

The present case is distinguished by several features: (1) a significant diagnostic delay of approximately one year, during which the patient was initially evaluated by a dermatologist and subsequently referred through surgical departments before a dental origin was suspected; (2) the occurrence in a 13-year-old with a fully erupted permanent mandibular first molar (tooth 36), highlighting age-specific challenges in immature permanent teeth with relatively wide canals and thin dentin walls; (3) the presence of both intraoral and extraoral sinus tracts simultaneously; and (4) a complete 6-month follow-up demonstrating progressive periapical healing and spontaneous closure of the cutaneous lesion without any surgical intervention or scar revision. These elements underscore the importance of early dental referral and the efficacy of conservative nonsurgical endodontic management in preventing unnecessary procedures and facial scarring in pediatric patients. While isolated pediatric cases of odontogenic cutaneous sinus tracts have been reported, most describe management involving surgical excision or scar revision of the cutaneous tract. To our knowledge, this case is among the few documenting complete spontaneous closure of a sinus tract present for more than half a year using nonsurgical endodontic treatment alone, with the simultaneous presence of an intraoral and an extraoral tract and no adjunctive surgical, antibiotic, or scar-revision procedure of any kind.

In the scientific literature, there are a small number of described clinical cases of patients with odontogenic cutaneous sinus tract. Each of them is associated with specific clinical findings and therapeutic solutions. The individual characteristics of the patients and their different dental and actual age are factors for diversity in the approaches. Therefore, we set ourselves the task of presenting a protracted clinical case of odontogenic cutaneous sinus tract, which successful resolution by nonsurgical endodontic therapy alone, without surgical excision of the cutaneous lesion.

## 2. Case Presentation

### 2.1. Study Design

This study presents a single clinical case of nonsurgical endodontic management of a mandibular first molar associated with an odontogenic cutaneous sinus tract in a pediatric patient. The treatment protocol followed standard endodontic principles, with emphasis on infection control, chemomechanical debridement, and intracanal disinfection.

### 2.2. Clinical Examination and Diagnosis

#### 2.2.1. Patient Information

A 13-year-old boy was referred to the Department of Pediatric Dentistry, Medical University of Sofia, for endodontic management of tooth 36. The patient was referred from the Department of Oral and Maxillofacial Surgery for endodontic treatment of tooth 36. The family initially sought care at a surgical department due to a cutaneous lesion on the left side of the face. The patient reported intermittent pain and discomfort during mastication in a tooth on the left side.

#### 2.2.2. History of the Present Complaint

The patient and his father reported the appearance of a small nodule on the left side of the face approximately one year earlier, which gradually enlarged over time. They first consulted a dermatologist. The patient was subsequently referred to a surgeon who directed him to a maxillofacial surgeon. Radiographic examination revealed a periapical inflammatory lesion associated with tooth 36. The dentition was poorly maintained, and palpation showed no evidence of lymphangitis. The patient was referred to the pediatric dentistry specialist for endodontic treatment of tooth 36.

#### 2.2.3. Extraoral Status

Facial symmetry was preserved. The skin of the face and visible body parts showed normal color and turgor, and visible mucous membranes were of normal appearance. On the left side of the face, in the projection of the inferior border of the mandible, a cutaneous lesion measuring approximately 8 × 6 mm, slightly elevated (~3 mm) present (Figure 1A). No suppuration was observed. No enlarged lymph nodes were palpable.

#### 2.2.4. Intraoral Status

The oral mucosa was normal and pale pink. The tongue was pale pink without mucosal lesions. The hard and soft palate were of normal pale-pink color and without lesions. The patient presented with mixed dentition, with most permanent teeth erupted. Retained primary teeth were noted (teeth 53, 84, and 85); extraction of these teeth was recommended and was included in the overall treatment plan. A fistula opening was observed in the gingival area between teeth 35 and 36 (Figure 1B).

Pulp vitality testing was not performed on tooth 36 due to the clear clinical and radiographic signs of pulpal necrosis, including the presence of a cutaneous sinus tract, intraoral fistula, large periapical radiolucency, and purulent exudate observed upon access. Electric pulp testing (EPT) and thermal testing are generally recommended in doubtful cases. However, in teeth with obvious necrotic pulp and established sinus tracts, these tests often provide limited additional diagnostic value and were therefore omitted. Adjacent teeth, including tooth 35 and the retained primary molars, were examined clinically (percussion, palpation, and visual/radiographic inspection) and showed no signs of caries, mobility, or periapical pathology, excluding them as alternative sources of infection. The persistent primary teeth 53, 84, and 85 were also evaluated; extraction of these teeth was recommended and was included in the overall treatment plan, and they were extracted during the course of treatment.

#### 2.2.5. Radiographic Examination

A periapical radiolucent lesion was evident in the region of tooth 36, involving both mesial and distal roots (Figure 1C). Secondary caries was evident beneath the existing restoration. The tooth showed a defective mesial restoration with secondary caries. A sinus tract was traceable between teeth 35 and 36. The tooth was tender to percussion. Gutta-percha point tracing of the sinus tract was not performed in this case, as the intraoral fistula and the periapical radiolucency on the periapical radiograph already provided a clear, unambiguous radiographic correlation with tooth 36. Cone-beam computed tomography (CBCT) was considered but judged unnecessary, since the two-dimensional periapical and panoramic radiographs were sufficient to localize the lesion, define its extent, and plan nonsurgical treatment. No microbiological sampling or culture was performed, as treatment was based on standard empirical chemomechanical disinfection protocols for clinically and radiographically diagnosed pulpal necrosis with an associated sinus tract, consistent with routine endodontic practice.

This figure provides the initial clinical and radiographic documentation. It highlights the location and appearance of the extraoral sinus tract on the left mandibular region and correlates it with the radiographic evidence of a necrotic mandibular first molar (tooth 36) with periapical pathology, thus establishing the link between the dental infection and the facial lesion.

Summary of findings and diagnosis: Clinical and radiographic examination revealed pulpal necrosis and chronic apical periodontitis of tooth 36 associated with both intraoral and extraoral sinus tracts. The definitive diagnosis was chronic apical periodontitis of tooth 36 with odontogenic cutaneous sinus tract. The proposed treatment plan—nonsurgical root canal treatment of tooth 36 with observation of the cutaneous lesion—was discussed with the patient and his parents, who provided informed consent.

### 2.3. Treatment Plan

The treatment plan consisted of nonsurgical endodontic therapy of tooth 36 and clinical observation of the cutaneous lesion. Before treatment initiation, the diagnosis, prognosis, and available treatment options were discussed in detail with the patient and his parents. The proposed nonsurgical endodontic treatment aimed to preserve tooth 36 and eliminate the source of infection while allowing spontaneous healing of the cutaneous sinus tract. Extraction of tooth 36 was also presented as an alternative treatment option. The potential benefits, risks, and limitations of each approach were explained, including the possibility of treatment failure and the need for additional interventions if healing was not achieved. After considering the available options, the patient and his parents elected to proceed with conservative nonsurgical endodontic treatment. Written informed consent was obtained before treatment was initiated.

At the first visit, chemomechanical debridement was performed under rubber dam isolation using 2.5% sodium hypochlorite irrigation and calcium hydroxide dressing. At the second visit, persistent exudation from the mesial canals required re-instrumentation and replacement of the intracanal medicament. At the third visit, when the canals were dry and asymptomatic, obturation was completed with gutta-percha and sealer, followed by definitive restoration. All procedures were performed under strict aseptic conditions using rubber dam isolation. In addition to the endodontic treatment of tooth 36, the persistent primary teeth 53, 84, and 85 were extracted during the course of treatment, as previously recommended and planned.

### 2.4. First Visit

After rubber dam isolation, the defective restoration and secondary caries were completely removed. An endodontic access cavity was prepared under magnification. A putrid odor and purulent exudate were noted upon opening. Working length was determined using an electronic apex locator and confirmed radiographically. Chemomechanical debridement was performed with stainless-steel K-files up to size 30/0.04 taper using a step-back technique. Copious irrigation with 2.5% NaOCl (heated to 37 °C) was delivered between files using a 30-gauge side-vented needle, with a total volume of approximately 20 mL per canal. A final irrigation sequence included 17% EDTA for 1 min to remove the smear layer, followed by a saline rinse. Due to persistent exudation, calcium hydroxide paste was placed as an intracanal medicament for its antimicrobial and tissue-dissolving properties, which are particularly beneficial in immature permanent teeth with wide canals. The access cavity was temporized with a sterile cotton pellet and temporary filling.

### 2.5. Second Visit (After 10 Days)

The tooth was re-isolated with a rubber dam, and the temporary restoration was removed. No putrid odor was detected; however, exudate persisted from the mesial canals. The canals were re-instrumented using K-files up to size 30/0.04 and irrigated with 2.5% NaOCl. A fresh calcium hydroxide intracanal dressing was placed, followed by a sterile cotton pellet and temporary restoration.

### 2.6. Third Visit (After 10 Days)

The tooth was isolated with a rubber dam, and the temporary restoration was removed. The canals were irrigated with 2.5% NaOCl and physiological saline. After drying, no purulent exudate was observed. After confirmation of a dry and asymptomatic canal system, obturation was performed using gutta-percha and an endodontic sealer. A glass ionomer cement base was placed, followed by a temporary restoration. A control periapical radiograph was scheduled.

### 2.7. Fourth Visit

The tooth was isolated with a rubber dam. The glass ionomer base was refined, and the definitive composite restoration was placed. Follow-up periapical radiographs were obtained.

### 2.8. Management of the Sinus Tract

No surgical intervention or direct treatment of the cutaneous sinus tract was performed. Resolution was expected following elimination of the intracanal infection.

### 2.9. Follow Up Protocol and Outcome

Clinical and radiographic follow-up examinations were conducted at 2 weeks, 1 month, 3 months, and 6 months post-treatment (Figure 2). Healing was assessed based on the resolution of the extraoral lesion, the absence of clinical symptoms, and radiographic evidence of periapical bone regeneration (Figure 2).

Serial radiographs and clinical photographs document the course of healing, showing progressive reduction in the periapical radiolucency and gradual closure of the extraoral lesion after elimination of the odontogenic source. These images provide visual confirmation of the treatment outcome and support the diagnosis and management described in this report.

This figure demonstrates the longitudinal radiographic healing. By comparing the baseline image with post-treatment and 6-month follow-up radiographs, it visually confirms the progressive bone regeneration and reduction in the periapical radiolucency, directly correlating the success of the nonsurgical endodontic therapy with internal tissue healing.

Figure 3 presents a chronological sequence of the facial lesion’s appearance from baseline through 2 weeks, 1 month, 1.5 months, 3 months, and 6 months. It serves as visual proof of the gradual, spontaneous closure of the fistula, illustrating that the extraoral manifestation resolved sequentially following the elimination of the odontogenic source.

## 3. Discussion

This case demonstrates that odontogenic cutaneous sinus tracts in children may remain undiagnosed for prolonged periods when dental symptoms are minimal and the lesion is initially assessed as a dermatologic problem. The favorable outcome in this patient confirms that elimination of the intracanal infection alone can resolve both the periapical lesion and the extraoral tract, provided that diagnosis is timely and the tooth remains restorable. Conventional nonsurgical endodontic therapy alone led to complete resolution of the cutaneous lesion without the need for surgical excision.

The diagnosis of an odontogenic origin for the cutaneous sinus tract in this case was based on the clinical and radiographic findings directly associated with tooth 36, consistent with the pathogenesis described above [23,25,26,27]. Upon access cavity preparation, a putrid odor and purulent exudate were evident from the root canals, confirming pulpal necrosis and chronic suppurative apical periodontitis as the source of infection. The sinus tract was traced intraorally between teeth 35 and 36, and the preoperative radiographs revealed a large periapical radiolucency involving both roots of tooth 36, establishing a clear cause-and-effect relationship between the necrotic pulp of tooth 36 and the extraoral cutaneous lesion.

Histobacteriologic studies have demonstrated that teeth with chronic apical abscesses and sinus tracts often harbor complex infectious patterns, including bacteria in the apical root canal system and extraradicular biofilms on the outer root surface [26]. Chronic suppuration leads to bone resorption and fistulous tract formation, which may drain intraorally or, less commonly, externally. In pediatric patients, the mandibular-submandibular region is most commonly affected, with mandibular first molars and incisors being the teeth most frequently involved [21]. Dental caries remains the primary etiology, and the lack of overt symptoms often results in delayed diagnosis and mismanagement [20].

Nonsurgical root canal therapy is the treatment of choice for periapical lesions associated with sinus tracts in restorable teeth. Elimination of the infection through thorough chemomechanical debridement, disinfection, and obturation typically results in complete resolution of the sinus tract [20,21,28,29]. In the present case, this approach resulted in complete resolution of both the periapical lesion and the cutaneous sinus tract over 6 months, despite the infection having been present for approximately one year.

In a pediatric case series of 28 children, treatment consisted of either root canal therapy or extraction. Early intervention led to spontaneous healing without scarring, whereas delayed treatment (mean lesion duration 6.5 months) was associated with a 29% rate of surgical scar revision [21]. Similarly, our case achieved spontaneous healing without scarring despite a longer diagnostic delay of one year, supporting the importance of thorough nonsurgical management even in delayed presentations.

A clinical review of 37 cases found that 80% of teeth were treated endodontically, and all fistulas healed uneventfully following proper dental therapy without the need for cosmetic surgery; complete resolution occurred within eight weeks in 63% of cases [30]. Our findings are consistent with this review, as the cutaneous sinus tract in our patient resolved completely without any surgical or cosmetic intervention.

Nonsurgical treatment of teeth with large periapical lesions also achieves high success rates, with reported periapical healing rates ranging from approximately 67–89% depending on outcome criteria, lesion size, and the presence of a sinus tract [31,32,33]. The favorable outcome observed in our case, with progressive periapical healing and complete closure of the extraoral tract, is consistent with these previously reported success rates for nonsurgical management.

In children, odontogenic cutaneous sinus tracts differ from adults in lesion site, duration, and affected teeth, but etiology and treatment approach are similar [21]. Early diagnosis and treatment are critical to prevent scarring and unnecessary interventions, especially in patients with poor preventive dental care [20,21]. This case demonstrates that even after a prolonged period of infection, conservative nonsurgical endodontic therapy can lead to excellent clinical and esthetic outcomes.

Cutaneous sinus tracts of dental origin present diagnostic challenges [23]. Patients may lack dental symptoms or fail to associate them with the skin lesion, and the tract may open at a site distant from the offending tooth [34]. Misdiagnosis often leads to inappropriate management, including prolonged antibiotic therapy, surgical excision, and, in rare cases, radiotherapy [23]. While systemic antibiotics may temporarily reduce drainage, the sinus tract invariably recurs unless the dental source of infection is definitively addressed [25].

Despite the long duration of the lesion (approximately one year), nonsurgical root canal treatment resulted in complete resolution of both the periapical pathology and the cutaneous sinus tract without scarring. Based on this favorable outcome and supported by the literature, we strongly recommend that nonsurgical endodontic therapy should be the first-line treatment for restorable teeth with odontogenic cutaneous sinus tracts in children. Extraction should be reserved for cases with extensive coronal destruction, poor restorability, or when endodontic treatment fails. In pediatric patients, tooth preservation is particularly important for maintaining arch integrity, function, and esthetics during growth.

From our clinical experience, the main diagnostic challenge in this case was the prolonged delay before a dental origin was suspected, as the patient was initially referred to dermatology and oral-maxillofacial surgery departments. This highlights the importance of routine dental evaluation, including periapical and panoramic radiographs, in any child presenting with a persistent facial nodule or sinus tract in the mandibular region. The final diagnosis of an odontogenic cutaneous sinus tract secondary to pulpal necrosis of tooth 36 was confirmed by the presence of purulent exudate and putrid odor upon access, the radiographic periapical lesion, and the complete resolution of the extraoral tract after elimination of the intracanal infection. Differential diagnosis of a facial cutaneous sinus tract should include dermatological conditions such as pyogenic granuloma, furuncle, infected epidermal or pilar cyst, foreign body reaction, and, less commonly, neoplastic processes or chronic infections such as actinomycosis or osteomyelitis. In children, developmental anomalies may also be considered. Careful intraoral examination combined with radiographic tracing of the tract usually allows differentiation of the odontogenic origin.

The pathway of infection in these tracts is determined by the path of least resistance through soft tissues and the relationship of the tooth apex to adjacent muscle attachments. In the present case, the tract extended superior to the buccinator muscle attachment, resulting in a cutaneous fistula. Such tracts typically manifest as persistent papules or nodules with intermittent purulent discharge, often on the chin or mandibular region, and may produce skin dimpling due to fixation to underlying tissues.

This case highlights the importance of timely referral, accurate diagnosis, and meticulous chemomechanical debridement in immature permanent teeth. By addressing the periapical infection at its source, both periapical healing and spontaneous closure of the fistulous tract were achieved. Long-term radiographic and clinical follow-up (up to six months) confirmed sustained resolution and tooth survival, reinforcing that elimination of the odontogenic etiology is the cornerstone of management for cutaneous sinus tracts of dental origin.

## 4. Conclusions

This case differs from many routine reports because it involved a prolonged diagnostic delay in a child, initial consultation with non-dental specialists, an extraoral lesion in the mandibular region, and full resolution through nonsurgical endodontic treatment alone. These features emphasize the need for multidisciplinary awareness and prompt dental referral when facial skin lesions persist without clear dermatologic cause. This case demonstrates that nonsurgical endodontic therapy alone can achieve complete resolution of both periapical infection and associated extraoral sinus tracts, avoiding the need for surgical excision. Early recognition, accurate diagnosis, and appropriate dental management are essential to ensure favorable clinical outcomes, prevent scarring, and preserve the natural tooth in the dental arch.

## Figures and Tables

**Figure 1:**
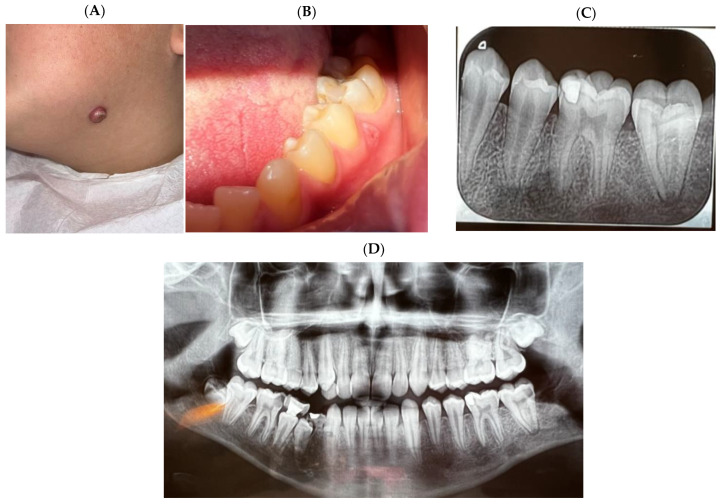
Initial case presentation. (**A**) Extraoral view showing a slightly elevated cutaneous nodule measuring approximately 8 × 6 mm located on the left mandibular border. (**B**) Intraoral view demonstrating the fistula opening in the gingival sulcus between teeth 35 and 36. (**C**) Preoperative periapical radiograph revealing a large periapical radiolucency involving both mesial and distal roots of tooth 36, secondary caries beneath the defective restoration, and a traceable sinus tract. (**D**) Preoperative panoramic radiograph showing the extent of the periapical lesion associated with tooth 36 and overall dentition status. These images collectively highlight the diagnostic challenge of an odontogenic cutaneous sinus tract in a pediatric patient presenting with minimal dental symptoms.

**Figure 2:**
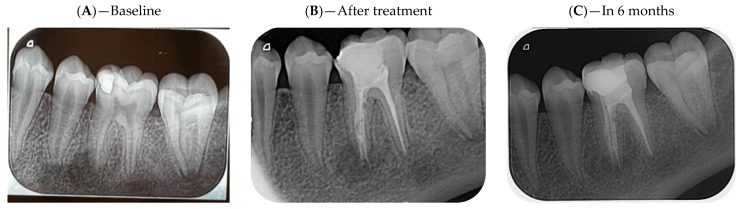
Periapical radiographs demonstrating progressive healing of the periapical lesion associated with tooth 36. (**A**) Baseline: Large periapical radiolucency involving the mesial and distal roots. (**B**) Immediately after obturation. (**C**) Six-month follow-up: Noticeable reduction in the size of the periapical radiolucency and evidence of bone regeneration. The radiographic sequence confirms successful periapical healing following nonsurgical endodontic therapy.

**Figure 3:**
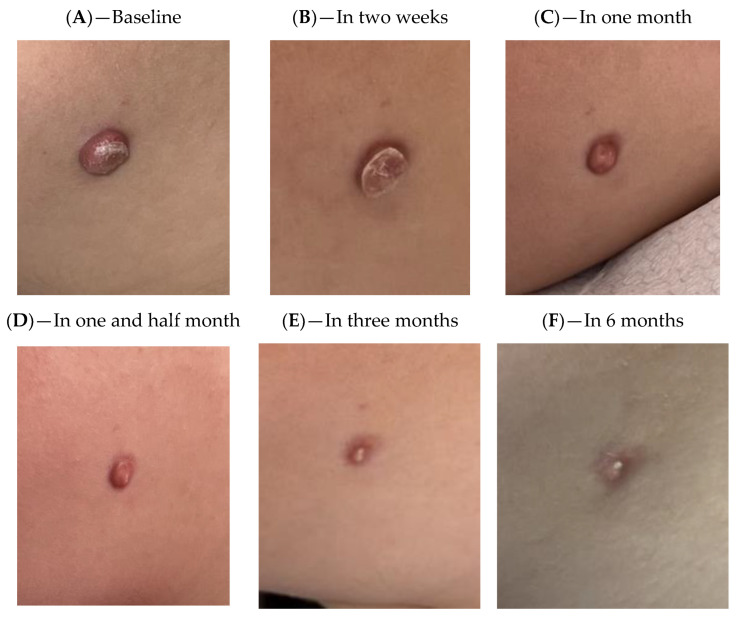
Clinical progression of the extraoral cutaneous sinus tract over 6 months. (**A**) Baseline: elevated nodule (~8 × 6 mm) with slight elevation on the left mandibular border. (**B**) Two weeks post-obturation: reduction in size and elevation. (**C**) One month: further flattening. (**D**) At 1.5 months, there was marked improvement. (**E**) Three months: almost complete resolution with minimal residual dimpling. (**F**) Six months—almost complete spontaneous closure without scarring or surgical intervention.

## Data Availability

The original contributions presented in this study are included in the article. Further inquiries can be directed to the corresponding author.

## Abbreviations

The following abbreviations are used in this manuscript:

NaOCl	Sodium hypochlorite
EDTA	Ethylenediaminetetraacetic acid
